# CBD for pets: navigating quality assurance, safety standards, and marketing strategies

**DOI:** 10.1186/s42238-024-00257-5

**Published:** 2025-01-23

**Authors:** Hannah Rideout, Alasdair J. C. Cook, Anthony D. Whetton

**Affiliations:** 1 vHive, School of Veterinary Medicine, University of Surrey, Guildford, GU2 7AL UK; 2 School of Biosciences, University of Surrey, Guildford, GU2 7HX UK

**Keywords:** CBD, Cannabidiol, Animal health, Canine, Feline, Disease

## Abstract

As the human cannabinoid (CBD) market grows, there is an inevitable transfer of the same or similar products into the veterinary sector. Advances in veterinary medicine and care of companion animals has led to extended life expectancy and consequently, there is an increased incidence of age-related chronic conditions that compromise quality of life. CBD products may alleviate these conditions. Research into CBD for companion animal species is on the rise, however, we found that there are no licensed veterinary CBD products available in the market due to a lack of appropriate testing and/or data. Here we outline the data that is available and show that the regulatory, and safety considerations around these products needs further consideration and this encompasses many products currently available on the market. Changes in regulations and further research for quality assurance are paramount to distribution of safe and applicable products for companion animals.

## Introduction

Life expectancy of pets, and popularity of companion animal ownership are growing. Therefore, research into ways to improve quality of life in our ageing pets is required. The development of novel pharmaceuticals and supplements is an approach with merit given what we have learned about diet and longevity in humans. Research has demonstrated that cannabidiol (CBD) compounds have a role in pain management, seizure treatment and improving overall wellness in treating human disease. Evidence also suggests that there may be similar value of CBD compounds for companion animals. However, current over-the-counter products are being sold and distributed in an unregulated environment. Here we discuss quality assurance, safety and efficacy matters for consideration by companion animal guardians and regulatory bodies.

### CBD and THC, two cannabinoids with markedly different effects

*Cannabis sativa*, a herbaceous plant species, contains over 120 phytocannabinoids; the two most abundant are Δ^9^-tetrahydrocannabinol (THC) and cannabidiol (CBD) (Ni et al. 2023). These compounds have similar structures that are reported to elicit different effects (Pintori et al. 2023). THC targets cannabinoid receptor 1 (CB1) whereas CBD has a low affinity for CB1, producing its effects at least in part, through the CB2 receptor (Stella 2023; Maccarrone et al. 2023). Both compounds are reported to produce potential curative effects for cancer, neurodegenerative disorders, and diabetes (Izzo and Camilleri 2009). Marijuana and hemp are two strains of *Cannabis sativa*, the former is primarily cultivated due to its high THC concentration responsible for the ‘high’ feeling associated with cannabis use, whereas hemp contains a high CBD concentration, alongside extremely low levels of THC. As THC has toxic effects in companion animals, here we only consider the use of CBD.

### The market for CBD in human health and wellbeing

It can be said that where the human medicinal compound market goes, the companion animal veterinary market will follow. Thus, we consider the growth of CBD use in humans. From 2018–2021 sales of CBD herbal supplements grew by 300% in the US, making it the top selling natural ingredient in the herbal supplement market (Li et al. 2021). In 2023, the global cannabidiol market was valued at approximately US$7.71 billion with an expected growth of 15.8% by 2030 (Grand View Research 2023). The cannabidiol market is becoming increasingly competitive with the introduction of online sales widening the prospective sales base. Also, the growing market encourages the appearance of new CBD-based companies, further boosting trade. Online marketing and promotions of the potential benefits of these products drives consumers to purchase independent of price (Faizullabhoy and Wani 2022). Additionally, the changing regulation of cannabis and cannabis-based products globally, and a switch in categorisation of CBD products from herbal remedies to pharmaceuticals, as seen with Arvisol (a patented CBD oral tablet), are anticipated to drive growth in consumer numbers (Grand View Research 2023). Several US states have passed laws that remove state restrictions on the medical use of cannabis and given the publicity around use of CBD to treat seizures, these compounds may be viewed as of benefit in companion animals.

Regionally, North America dominates the CBD market with the growth attributed to a higher number of health-conscious individuals, the presence of more manufacturers, and the approval of the US Farm Bill 2018 (assurance that any cannabinoid derived from hemp is legal if produced in a manner consistent with the Farm Bill and by a licensed grower) (Hudak 2018). Furthermore, Europe is predicted to have increased growth due to enhanced benefit awareness, availability, and affordability; with Asia also a growing market given it’s established production facilities, and export markets making it a major competitor for US, Canadian and EU hemp production (Grand View Research 2023).

It is important to note that unlike the human CBD market that receives research financial support, it is commercial interests that drive veterinary drug development, with companies only investing where they see viable commercial returns (HPRA 2014). Consequently, research towards the global veterinary medicine market is lacking compared to the human medicine market.

### Human CBD products available and their relation to defined medicines

A medicinal product is defined as “Any substance or combination of substances presented as having properties for treating or preventing disease in human beings”, and “Any substance or combination of substances which may be used in, or administered to, human beings, either with a view to restoring, correcting or modifying physiological functions by exerting a pharmacological, immunological or metabolic action, or to making a medical diagnosis” (MHRA 2020). Legally, a product can be marketed as ‘medicine’ if it adheres to the definition of a medicinal product, has all available evidence assessed, and has adhered to relevant court precedents. Similarly, ‘therapeutics’ are defined broadly as products used in humans to prevent, diagnose, cure, or alleviate a disease, ailment, defect, or injury that influences, inhibits, or modifies a physiological process (TGA 2022).

A CBD-based product approved for medical use in humans (Table 1) is Sativex (data sheet) which is equal parts CBD and THC (used to treat neuropathic pain and spasms in Multiple Sclerosis patients). It is approved in various countries including the UK and EU, but not the US as they state it does not meet the primary criteria outlined above. However, Epidiolex® (data sheet) is approved in the US, as well as other countries for the treatment of seizures.

**Table 1:**
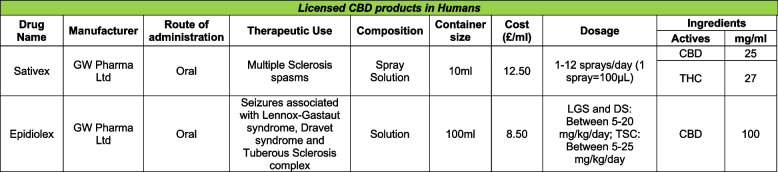
Licensed CBD products in humans

Other marketed products of CBD compounds for human use are diverse and include oils, gel capsules, edibles, vapes and topical creams, the most popular form being edibles; under US law, CBD products are classified as legal if the THC content is ≤ 0.3% (Li et al. 2021; Wheeler et al. 2020). In the US, 38% of CBD consumers reported using the products for general health and wellness and 62% for pain relief and better sleep (Corroon and Phillips 2018). The product range available spans various claims, from inclusion of CBD to being a “hemp extract” to containing “activated cannabinoids”. Many commercial products have been found to contain different inclusions levels than the label claims, with 14% showing a CBD concentration level of only 0.0001% w/w (Miller et al. 2022).

It is also important to note with respect to the above guidelines for therapeutics, most CBD products on the market are sold as supplements (NHS 2023). However, in 2023, the FDA stated that nothing called into question its current conclusions that THC and CBD products are excluded from their definition of a dietary supplement (A dietary supplement is an ingested product that contains a "dietary ingredient" intended to supplement the diet, e.g., vitamins and minerals; herbs and metabolites), maintaining that CBD is an approved drug which prevents its addition to food and beverages. CBD is classified as a ‘novel food’ in the UK and EU. As such, it cannot be legally included in a food supplement until a safety assessment has been completed and the novel food has been authorised (Food Standards Agency 2023). Furthermore, any deviation within the recipe requires a new authorisation process for the supplement. Interestingly, the European Food Safety Authorisation (EFSA) have published a statement following their confirmation of CBD as a novel food, stating that there are clear knowledge gaps that must be addressed before the safety of CBD can be concluded (EFSA, et al. 2022). Hemp does not fall under the same regulations as CBD, so can be included in food supplements, providing certain criteria are met (e.g., the product is not contaminated with other cannabinoids) (Chartered Trading Standards Institute 2024).

### CBDs and a consideration of companion animal health

In 2021, the global pet care market has an estimated value of US$150.67 billion, predicted to increase by 5.1% by 2030 (Grand View Research 2024). The rise is associated with increased anthropomorphism and greater care taken of companion animals, plus greater consumer spending on pets in the home. Also, there appears to be a definite shift in age of pet owners as households owned by younger generations (pre-millennials) now make up 60% of owners, and they are demonstrating more interest in companion animal preventative care measures (Grand View Research 2024).

Over half of the global population is estimated to own a pet, with dogs and cats being the most popular companions (Health for Animals 2022). In 2018, there were approximately 470 million dogs and 370 million cats globally, set to continue rising (Shahbandeh 2018). The life expectancies of dogs and cats vary between breeds and sexes (Willems et al. 2017). However, as a guideline, dogs live on average 11.75 (4.5–19) years (Teng et al. 2022), and cats 14 (9–17) years (O'Neill et al. 2014). Since 2013, the average life span of dogs has increased approximately 5.5% (Teng et al. 2022); and for cats, roughly 11% since 2009 (O'Neill et al. 2014). Ageing pets represent 30–40% of veterinary patients due to the increased risk of them developing chronic illnesses (Willems et al. 2017). As our pets are living longer, they are consequently experiencing increasing age-related problems and illnesses, as seen in humans. The most frequent age-related medical problems in dogs are skin conditions, orthopaedic issues (e.g., osteoarthritis), gastrointestinal issues, ENT issues (e.g., infections, deafness), cancer, neurological conditions, liver and kidney conditions, and endocrine disease (e.g., hypothyroidism, diabetes) (Nam et al. 2024). Similarly, older cats are often found to suffer with mobility issues (e.g., osteoarthritis), dental issues, endocrine disease (e.g., hyperthyroidism), hypertension, neurological conditions, organ failure (e.g., kidney, liver), and cognitive dysfunction syndrome (Sordo et al. 2020). As the popularity of companion animals grows, alongside the average life expectancy, more research efforts are focusing on improving the quality of life for ageing pets. One example of this is the development of new pharmaceuticals and supplements to diminish the negative effects of ageing. This will create a new market in aged pet care. There is a clear case that consumers will demand companion animal care to alleviate pain and improve wellness in the latter stages of their companion animal’s life, and questionnaires show clearly that the spending in this area can often be a significant portion of household income (HABRI 2021).

### Current disease orientated research findings in animals relating to CBD

Publication bias with respect to negative data in clinical studies is a well-known phenomenon (i.e. negative data is not published). Additionally, current research in this field is limited, with outcomes that are published detailing conflicting findings and being based on small sample sizes (Table 2). With that stated, our review of the literature detailed below generally shows a positive outlook for the future of CBD use in veterinary medicine. Further insights into how CBD can be used appropriately are crucial for optimal use of these compounds (Parker 2022).

**Table 2:** Peer reviewed studies on CBD effects on companion animals

Condition	Species	No of animals	Dosage	Objective	Result/Conclusion	Reference
Pruritis	Dogs	8	0.07–0.125 mg/kg twice daily	Examine the effects of CBD-containing hemp oil without THC as a supplemental treatment for Canine Atopic Dermatitis in dogs	CBD was well tolerated over a wide dose range, decreasing pruritic occurrence in dogs with CAD when given twice daily	(Mogi et al. 2022)
		32	2 mg/kg CBD/CBDA mix twice daily for 28 days	Determine if CBD/CBDA is an effective therapy for CAD	CBD/CBDA decreased pruritic occurrence as an adjunct therapy but did not decrease skin lesions associated with CAD in dogs	(Loewinger et al. 2022)
		15	N/A	Investigate the immunohistochemical expression of cannabinoid receptors in keratinocytes of healthy dogs and dogs with atopic dermatitis	Presence of cannabinoid receptors in healthy keratinocytes suggests a possible role of the endocannabinoid system in epidermal homeostasis. Potential therapeutic target for dogs with atopic dermatitis	(Chiocchetti et al. 2022)
Anxiety	Cats	10	4.0 mg/kg/day for 2 weeks	Examine the effects of CBD administration upon separation anxiety in healthy cats	The results suggest anxiety-reducing effects of CBD in cats	(Masataka. 2024)
	Dogs	16	25 mg CBD, trazodone (100 mg for 10–20 kg BW, 200 mg for 20.1–40 kg BW), combination of CBD and trazodone	Evaluate the influence of CBD on behavioural responses to fear-inducing stimuli in dogs	The results do not support anxiolytic effects of CBD in dogs given 1.4 mg/CBD/kg BW/day	(Morris et al. 2020)
		24	0-5 mg/CBD/kg BW/day	Determine the influence of CBD on the daily activity of adult dogs	CBD does not impact the daily activity of adult dogs when supplemented with up to 4.5 mg/CBD/kg BW/day. May exert antipruritic effects	(Morris et al. 2021)
		24	5% CBD oil	Determine if CBD affects stress related behaviour in shelter dogs	No significant results	(Corsetti 2021)
		40	4 mg/kg	Evaluate the effect of a single dose of a THC-free broad-spectrum CBD distillate on measures of canine stress during separation and car travel	The mitigating effect of CBD treatment varied by measure and test, with some indicating a significant reduction in canine stress compared to the placebo group	(Hunt et al. 2023)
		19	4 mg/kg	Evaluate whether multiple doses of a THC-free CBD distillate over a period of 6 months could positively influence measures of stress in dogs	The mitigating effect of CBD treatment varied by measure, with cortisol, whining, lip licking, and qualitative behavioural ratings indicating a significant (P < 0.05) reduction in canine stress compared to the placebo group for at least one time point	(Flint et al. 2024)
Pain Relief/ Osteo-arthritis	Cats	22	4 mg/12 h for 15 days	Evaluate the clinical efficacy and safety of a commercially available CBD oral formulation as an adjunctive treatment for pain management of feline chronic gingivostomatitis (FCGS)	The protocol is safe since severe adverse effects and biochemical changes were not observed during the treatment period. This study suggests that the cats benefited from this treatment	(Coelho et al. 2023)
	Dogs	4	2 or 8 mg/kg	Determine basic oral pharmacokinetics, and assess safety and analgesic efficacy of a cannabidiol (CBD) based oil in dogs with osteoarthritis	Study suggests that 2 mg/kg of CBD twice daily can help increase comfort and activity in dogs with OA	(Gamble et al. 2018)
		10	2.4 mg/15 kg	Evaluate the efficacy of a new diet supplement in reducing chronic pain and improving mobility in dogs with Osteoarthritis	Results of a Generalized Linear Mixed Model (GLMM) on HCPI highlighted a significant reduction of pain scores at the end of the study	(Martello et al. 2019)
		21	2 mg/kg twice daily	Evaluate the efficacy of oral transmucosal CBD, in addition to a multimodal pharmacological treatment for chronic osteoarthritis-related pain in dogs	Pain Severity Score and Pain Interference Score was significantly lower in CBD than in Control group	(Brioschi et al. 2020)
		20	20-50 mg/day naked CBD or 20 mg/day liposomal CBD	Evaluate the safety and efficacy of CBD in a double-blind, placebo-controlled study in a spontaneous canine model	Administration of liposomally-encapsulated or high-dose naked CBD (but not low-dose naked CBD or placebo) was associated with significant improvements to quality of life	(Verrico et al. 2020)
Epilepsy	Dogs	26	2.5 mg/kg twice daily for 12 weeks	Assess the effect of oral CBD administration in addition to conventional antiepileptic treatment on seizure frequency in dogs with idiopathic epilepsy	A significant reduction in seizure frequency was achieved for dogs in the CBD group, however the proportion of responders was similar between groups	(McGrath et al. 2019)
		14	2 mg/kg twice daily for 12 weeks	Examine a small cohort in a pilot investigation using a CBD and CBDA-rich hemp product for the treatment of refractory epileptic seizures in dogs	The use of 2 mg/kg every 12 h of a CBD/CBDA-rich hemp extract can have benefits in reducing the incidence of epileptic seizures, when used concurrently with other anti-seizure medications	(Garcia et al. 2022)
	Cats	9	5 mg/kg	Describe the disposition of a single dose of a cannabidiol medication in healthy cats in both the fed and fasted state	The relative bioavailability of CBD shows a near 11-fold increase when administered in the fed state compared to the fasted state. Additionally, concentrations achieved at a dose of 5 mg/kg, may be sufficient to explore the therapeutic potential in cats with epilepsy	(Jukier et al. 2023)

The US Food and Drug Administration (FDA) state “FDA is aware of some cannabis products being marketed as animal health products. We want to stress that FDA has not approved cannabis for any use in animals, and the agency cannot ensure the safety or effectiveness of these products. For these reasons, the FDA cautions pet-owners against the use of such products and recommends that you talk with your veterinarian about appropriate treatment options for your pet”. The FDA also states that it has not directly received any reports of adverse events associated with animals given cannabis products; however, a study published by the University of Guelph found that the number of reports to the US animal poison control centre (APCC) of dogs with cannabis poisoning is rising (Howard-Azzeh et al. 2021). Nonetheless, as CBD products gain popularity in the human supplement market, naturally the potential for application and market growth has transferred to companion animals. In 2017, US$ 8 million of CBD pet products were sold in the US, rising to $560 million by 2021 (Shahbandeh 2024b). As with the human market, North America holds the top spot for CBD product sales with Europe close behind, expecting sales to double to US$142 million by 2024 (Shahbandeh 2024b). The CBD pet care market is split between dogs (65%), horses (22%), cats (8%), and others (5%) (rabbits, small rodents and birds) (Shahbandeh 2024c). Despite the size of the pet CBD market, no licensed products are currently available for veterinary use, as there are insufficient data surrounding safety and dosage rates (VMD 2022).

In the US, it is illegal for vets to prescribe, dispense or recommend cannabis or its products for animals (AVMA 2019). However, in the UK and EU, vets are permitted to prescribe CBD products to pets through the cascade (a legislative provision in the Veterinary Medicines Regulations that allows a veterinary surgeon to prescribe unauthorised medicines that would not otherwise be permitted), although it is an offense for CBD products to be given to pets without a prescription (VMD 2022). As in humans, dosage behaviour means that owners administering CBD products to their pets are likely to guess dosage based on insufficient evidence and risk adverse reactions. There is also a concern that CBD may display “bell shaped dose response curves” in that too much of the drug has lesser or no effect compared to the optimal dose. The idea that safety in humans provides data for safety in companion animals is not supportive of licencing due to higher toxicity risk to THC in pets. Furthermore, unlicensed products go through much less rigorous testing, if any, and they may contain contaminants toxic to pets (e.g., high selenium levels); currently there are no proven benefits associated with these products (Summerhill Vets 2024).

Pet owners across the US, EU and Canada have been surveyed regarding their use of CBD products for their pets (Kogan et al. 2019; Tomsič et al. 2022; Corsato Alverenga et al. 2023). In all cases, the majority of owners obtained their information from online sources, with a much lower proportion consulting veterinarians. As a result, there appeared to be a lack of knowledge regarding safe dosage rates and what type of CBD they were administering to their pet. Across the studies, most owners reported using the products mainly as supportive therapies, followed by cases of resorting to CBD when conventional treatments failed. Approximately 1 in 20 British pet owners gave CBD products to their pets in 2022 (Hodges et al. 2022), and approximately 28% of US owners have previously administered CBD to their pet (Corsato Alverenga et al. 2023). It can be argued that this situation is not favourable for optimal treatment strategies for companion animals and value for money for the pet guardian. Further research on patient benefit with respect to conditions in ageing is needed to overcome such concerns.

However, within the present market this is not easily achieved. There are thousands of cannabinoid-containing products available in the pet care market in the forms of oils, edibles, and topicals. Given that CBD is not licenced for medicinal use, the products are sold adhering to packaging and labelling laws: for example, all CBD products marketed for animals need to be marketed as ‘supplements’ to differentiate them from medicines; it must state on the packaging that the product contains CBD, as what compounds, and how much (mg/kg); certain claims such as ‘preventative’ cannot be made unless the product is marked as medicinal; and lastly, the label must provide recommended dosage levels and a toxicity warning (GOV UK 1996).

There is concern regarding the quality of available CBD products on the market, especially for pets. A 2022 review of the cannabinoid market indicated that consumers’ biggest concerns are about the product purity (42%), price (38%), whether the product is Food Standards Agency (FSA) approved (38%), and poor labelling (31%) (Hodges et al. 2022). Additionally, information deemed to mean that the product is a trustworthy item to consumers in descending order are price, inclusion of natural ingredients, clear labelling of ingredients, and clarity on product origins. Purity, pricing and labelling of over-the-counter CBD products is currently un-regulated, therefore, many products do not meet consumer expectations (Table 3), though the market continues to flourish. As CBD is a (complementary) medicinal product in the UK and EU it requires a Veterinary Medicines Directorate (VMD) or European Medicines Agency (EMA) marketing authorisation to be prescribed (Wessmann et al. 2021). Without this, selling, supplying, or advertising CBD-containing products to pets is an offense*.*In the UK, CBD products are prohibited for use for pets altogether, so much so that online retailers have been forced to cease sales within the UK. Nevertheless, CBD products are still purchased by UK customers. Evidence-based veterinary medicine data does not seem to influence consumer appetite for products in some quarters (Tomsič et al. 2022; Holst et al. 2024).

**Table 3:** A range of pet CBD products available online; information is as seen on the website. (Price conversions correct as of May 2024). Terminology used is as follows (Canine Arthritis Management 2021): Full/Broad spectrum, unique medicinal profile influenced by where and how the plant is grown, and extraction process. Certificate of Analysis (CoA) is required to determine exact composition; CBD-rich, provides no other information about what is in the product (including THC content); Hemp seed oil, made from cannabis but any benefits are attributed to omega-3 levels; CBD isolate, a single compound, labelling must explain where compound was derived (CoA); CBD oil, vague, required CoA for clarity of composition

*CBD Pet Products*								
**Product Name**	**Manufacturer**	**Route of Administration**	**Composition**	**Package size**	**Price per pack (£)**	**Dosage Instructions**	**Label Claim**	
							**CBD source as stated in Ingredients List**	**CBD inclusion (stated units)**
Full Spectrum CBD Oil	Norwich	Oral	Oil	10 ml	29.95	1 drop = 2.5 mg CBD. Less than 2 kg: 1 drop/day, 2.3- 9 kg:1–2 drops/day, 9-23 kg: 3–6 drops/day, 23 kg + : 5–8 drops/day	Full Spectrum, Rich CBD Oil	500 mg
CBD	Mary Jane	Oral	Oil	10 ml	20.00	Up to 9 kg: 1–4 drops, 9-18 kg: 2–8 drops, over 18 kg: 4- 20 drops	CBD isolate	2%
CBD Oil	Hemx	Oral	Oil	30 ml	250 mg: 22.50, 500 mg: 30.95, 1000 mg: 47.95	0.5 mg CBD/kg BW	Full Spectrum CBD Oil	250 mg bottle: one drop = 0.28 mg; 500 mg bottle: one drop = 0.56 mg; 1000 mg bottle: 1 drop = 1.12 mg
CBD for Cats 2%	Cibapet	Oral	Oil	10 ml	14.04	1–2 drops 2–3 times a day	Hemp extract	200 mg
CBD for Cats 4%	Cibapet	Oral	Oil	10 ml	25.11	1–2 drops 2–3 times a day	Hemp extract	400 mg
CBD for Dogs 2%	Cibapet	Oral	Oil	10 ml	14.04	2–3 drops/10 kg/day	Hemp extract	200 mg
CBD for Dogs 4%	Cibapet	Oral	Oil	10 ml	25.10	2–3 drops/10 kg/day	Hemp extract	400 mg
CBD pastilles for Cats	Cibapet	Oral	Tablet	100 tablets	12.34	Not stated	Not stated	130 mg
CBD pastilles for Dogs	Cibapet	Oral	Tablet	55 tablets	16.60	2–6 tablets/10 kg/day	Not stated	86 mg
CBD oil for cats 2.5%	Renova	Oral	Oil	30 ml	25.74	Up to 2–3 drops, 3 times a day	Hemp extract	750 mg
CBD oil for dogs 2.5%	Renova	Oral	Oil	30 ml	11.78	Up to 3–4 drops, 3 times a day	Hemp extract	750 mg
CBD oil 5%	Renova	Oral	Oil	30 ml	48.43	Up to 3–4 drops, 3 times a day	Hemp extract	1500 mg
Pet Tincture (Dogs)	CBD FX	Oral	Oil	30 ml	23.60	1/2 dropper twice daily	CBD-rich hemp oil	250 mg
Pet Tincture (Cats)	CBD FX	Oral	Oil	30 ml	23.60	1/2 dropper twice daily	CBD-rich hemp oil	250 mg
CBD Calm pet oil	Bee(PETS)	Oral	Oil	30 ml	25.45	0.1 mg/kg every 12 h	Not stated	300 mg
CBD relief pet oil	Bee(PETS)	Oral	Oil	30 ml	25.45	0.1 mg/kg every 12 h	Not stated	150 mg
Stress Releaf	Pet Releaf	Oral	Oil	30 ml	35.40	Cat: 1-15lbs = 0.5 ml/day, over 15lbs = 1 ml/day. Dog: 1- 25lbs = 0.75 ml/day, 26-50lbs = 1.5 ml/day, 51- 75lbs = 2 ml/day, 76-100lbs = 2.75 ml/day	Organic Full-Spectrum CBD Oil	300 mg
Aceite de CBD	Dr. Green	Oral	Oil	10 ml	23.00	2–4 drops per day	Hemp extract	2.50%
CBD Capsules for pets	Hemp Heros	Oral	Oil	30 capsules	45.95	1 per day	Whole Hemp	25 mg
							THC	0.20%
Topical Cream	Treatibles	Topical	Cream	30 ml	18.89	Place a small amount on affected area every 4–8 h as needed	Hemp Oil	60 mg
Crème Apaisante	Bezzzen	Topical	Cream	118 ml	33.89	1 application every 4–6 h	Dimethicone Cannabidiol	3.50%
Tanacan Pet Cream	Herosan	Topical	Cream	30 ml	25.44	Apply as needed	CBD Premium whole extract	60 mg
Calming and Soothing Gel Transdermal Pen	CBD Pet Care	Topical	Gel	25 oz	31.46	1 pump = 2 mg CBD. Apply 1 pump/20lbs up to three times a day	Broad Spectrum Hemp Oil	100 mg
Hemp Oil	Primary Pets Premium Pet supplies	Oral	Gel Capsules	120 capsules	13.99	Up to 10 kg: 1 capsule every other day, 10-20 kg: 1 capsule every other day, 20-40 kg: 1 capsule per day, over 40 kg: 1 capsule per day	Hemp seed oil	1000 mg
CBD + CBDa Feline Paste	ElleVet	Oral	Paste	600 mg	62.13	5–15 lbs: 1 g twice daily, 16 + lbs: 2 g twice daily	Complete Spectrum Proprietary Hemp Oil	10 mg

It is recommended to ask for veterinary advice on the use of CBD products, however, in the US it is illegal in some states for vets to advise on prohibited substances such as CBD. Hemp is the only source legal to supply CBD in the US. Previously, hemp and CBD were federally illegal in the US as pet food ingredients (treats included) though changes to the Farm Bill (2018) moved hemp from schedule 1 to schedule 2 under the Controlled Substances Act to facilitate research in dogs (Corsato Alverenga et al. 2023). Additionally, as all states have their own individual laws about CBD products; this causes further legal issues and barriers for selling between states. Nonetheless the market remains buoyant for use of CBD products in companion animals, with vendors advertising, often appropriately, in making no claims for CBD beneficial effects.

It can be argued that there is a market opportunity for products that are based on hard scientific research with demonstrated benefit in a CBD product, that outstrips the conjecture often found associated with other products. Validity based on veterinary medicine research would certainly meet the consumer concerns and aspirations detailed above.

#### Tolerability of CBD in companion animals

Recent studies suggest that a daily dose of 4 mg/kg/day is well tolerated in healthy dogs and cats (Bradley et al. 2022; Coltherd et al. 2024). Evidence in the literature has shown that CBD administration to dogs is generally safe and well tolerated for short term use. Adverse effects such as loose stools and vomiting have been documented to affect 0.45–3.3% of dogs receiving CBD (Deabold et al. 2019; McGrath et al. 2018). Studies looking at serum chemistry in dogs largely report no significant alterations, but some have highlighted that liver enzymes, such as alkaline phosphatase (ALP), can become elevated (Gamble et al. 2018; McGrath et al. 2019; Deabold et al. 2019). Due to the complex nature of ALP level regulation, and the fact that age, stress, and certain medications (e.g., corticosteroids) can increase ALP levels, this warrants further investigation. Furthermore, without a concurrent significant rise in alanine transaminase (ALT) to support an indication of hepatocellular damage, the significance of these observations is uncertain. Consequently, controlled long term studies should be inclusive of a protocol for monitoring blood borne liver enzymes (Gamble et al. 2018).

One of the main safety concerns in companion animals appears to be the inclusion of THC. In randomized controlled trials, THC containing compounds produced more severe side effects in dogs than those in the control group, when tested for safety in escalating doses. In contrast to this CBD side effects were found to be mild even at high doses (640 mg) (Vaughn et al. 2020). THC causes toxicosis in dogs and cats and may present clinically as urinary incontinence, disorientation, hyperesthesia, bradycardia, diarrhoea, respiratory depression, twitching, and vomiting (Amissah et al. 2022). As THC is metabolised slowly by the liver of pets, clinical signs may only appear several hours post-ingestion (Chicoine et al. 2020). Concerningly, one US study found the most common source of toxicosis-causing compounds by vets are products from government-regulated producers (Amissah et al. 2022). In this review we are concerned with CBD which generally appears to only have mild side effects in dogs. Administration of CBD oil (62 mg/kg), THC oil (49 mg/kg), and a CBD: THC blend (12:8 mg/kg) were tested for safety in dogs, with all test groups showing mild adverse effects (lethargy, hypothermia) and some showing severe effects (neurological symptoms) (Vaughn et al. 2020).

Safety and tolerance data are largely un-reported in cats, with one study published in 2021 observing mild side effects (hypothermia, lethargy) when testing maximum doses of CBD (30.5 mg/kg), THC (41.5 mg/kg), and CBD: THC blend (13:8.4 mg/kg) (Kulpa et al. 2021). Another study tested doses of CBD oil ranging from 2.5-80 mg/kg where side effects (head shaking, excess salivation) were also found at levels ≤ 40 mg/kg (Rozental et al. 2023).

**Fig. 1:**
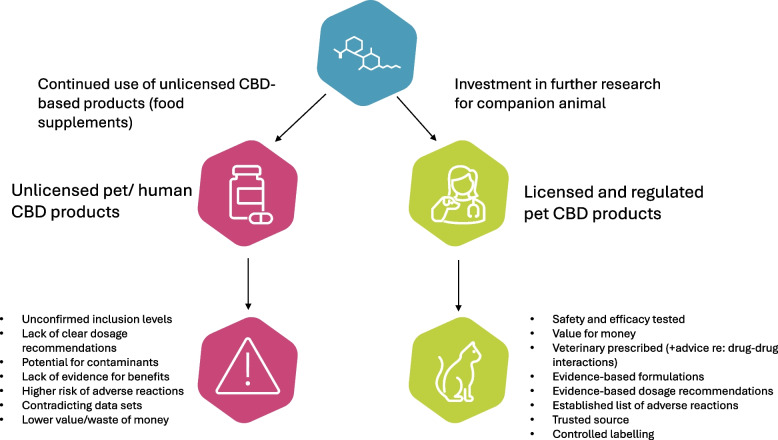
How the pet market may benefit from further research into licensed and regulated CBD-based drug development

Overall, to establish a market for a scientifically backed CBD-based product range, there is a definite need for further research into the use of CBD in both humans and animals to establish safety, tolerance levels, toxicity, optimum routes of administration, and further information regarding the metabolism and interactions within the body (Fig. 1).

## Discussion

Human health research shows that CBD can benefit many ailments, from stress to anxiety, depression, cardiovascular and neurological conditions. Companion animal research is showing similar outcomes with the evidence outlined in this review providing support for significant benefits of CBD compounds for some companion species already. Clearly, the ageing companion animal is another population who could, and should, be considered as potential recipients of beneficial treatments using CBD. However, due to limited evidence regarding the long-term safety of CBD in companion animals, further research is needed in this area.

Label accuracy and contamination concerns of CBD products in the marketplace have been abundantly published. Given that hemp is a non-regulated ingredient within the industry one would assume it is safe; however, it has long been reported as a bio-accumulator of toxic elements, being used for phytoremediation of contaminated soil (Linger et al. 2002). One study assessed 133 CBD products on the market and found 23% to contain one or more toxic elements above the limit of quantification (LOQ) including arsenic, cadmium, and lead (Dubrow et al. 2021). A similar study detected heavy metals in 22% of products tested, also detecting levels of pesticides and solvents, some of which at levels which violate regulatory thresholds (Gidal et al. 2024). In all studies analysing heavy metal contaminants, lead was the most prevalent (Wakshlag et al. 2020). In unregulated human CBD products tested for THC concentration, 65% contained levels of THC above LOQ, with 5 labelled specifically as ‘THC-Free’ containing detectable levels (Johnson et al. 2022). Similarly, in a range of CBD products tested for accuracy of CBD inclusion, only 31% were accurately labelled (Bonn-Miller et al. 2017). Not only do these studies provide concerns over quality assurance of products for humans, but even more so for the veterinary market as owners have reported using human products to treat their pets. A lack of accurate label information and quality assurance for these products provides a danger to consumers independent of species. There is a clear need for a representative investigation into the CBD market, to provide manufacturing and testing standards for these products.

All products (Table 2) make a point of “CBD" inclusion on the packaging, whether that be in the product name or as an advertisement on the bottle. However, many of the ingredient lists only vaguely state the source from which the CBD is originally derived i.e., cannabis, hemp derived CBD or whole organic hemp products. When vendors state inclusion levels, consumers are provided with either percentages or mg/bottle; it is not therefore certain, nor discernible in all cases we considered, at what inclusion level each ingredient is supplied per dose. Additionally, across all product labelling, dosage levels are vague, variable, overlapping and easy to misjudge. Some products could be alleged to be bypassing CBD-laws by marketing products containing CBD-containing plants such as hemp as 'Hemp oil' with the health benefits attributed to omega 3,6 + 9 rather than CBD. Also, in some cases, there is only a minor mention of CBD concentration in the fine print ingredients list.

The FDA does not approve CBD for use in animals. However, with some states also banning vets from advising owners on safe CBD use, this may also put pets at risk. In the UK and EU, the VMD and EMA consider veterinary products containing CBD to be veterinary medicines and so should be regulated as such. Therefore, CBD products marketed for animals are marketed as ‘supplements’ to differentiate them from medicines, and no CBD based products have been granted a UK or EU veterinary marketing authorisation. This review has shown how many over-the-counter supplements currently present risks to consumers, based on the lack of regulation, quality assurance, and clear labelling on their content. Therefore, we believe that the market cannot develop swiftly within an unregulated environment.

Given the positive outcomes in CBD research performed with respect to companion animal disease, there is a reason to believe that well-regulated products offer opportunities to improve animal health. However, currently there is an alarming lack of regulations and quality assurance throughout the products available on the market. We anticipate major developments in this field where reliable producers offer CBD containing preparations for improving health and welfare in companion animals. In order to facilitate this, more research is necessary, particularly in felines as they are vastly underrepresented throughout current studies (Table 2).

## Data Availability

No datasets were generated or analysed during the current study.
